# Etiquette for medical students’ email communication with faculty members: a single-institution study

**DOI:** 10.1186/s12909-016-0628-y

**Published:** 2016-04-27

**Authors:** Do-Hwan Kim, Hyun Bae Yoon, Dong-Mi Yoo, Sang-Min Lee, Hee-Yeon Jung, Seog Ju Kim, Jwa-Seop Shin, Seunghee Lee, Jae-Joon Yim

**Affiliations:** Department of Medical Education, Seoul National University College of Medicine, 103 Daehak-ro, Jongno-gu, Seoul, 03080 Republic of Korea; Department of Medical Education, College of Medicine, The Catholic University of Korea, Banpo-daero 222, Seocho-gu, Seoul, 06591 Republic of Korea; Division of Pulmonary and Critical Care Medicine, Department of Internal Medicine, Seoul National University College of Medicine, 103 Daehak-ro, Jongno-gu, Seoul, 03080 Republic of Korea; Department of Psychiatry and Behavioral Science, Seoul National University College of Medicine, 103 Daehak-ro, Jongno-gu, Seoul, 03080 Republic of Korea; Department of Psychiatry, Samsung Medical Center, Sungkyunkwan University School of Medicine, 81 Ilwon-dong, Kangnam-gu, Seoul, 06351 Republic of Korea; National Teacher Training Center for Health Personnel, Seoul National University College of Medicine, 71 Ihwajang-gil, Jongno-gu, Seoul, 03087 Republic of Korea; Department of Medical Science, Seoul National University College of Medicine, 103 Daehak-ro, Jongno-gu, Seoul, 03080 Republic of Korea

**Keywords:** Email writing, email etiquette, communication, graduate-entry program

## Abstract

**Background:**

Email is widely used as a means of communication between faculty members and students in medical education because of its practical and educational advantages. However, because of the distinctive nature of medical education, students’ inappropriate email etiquette may adversely affect their learning as well as faculty members’ perception of them. Little data on medical students’ competency in professional email writing is available; therefore, this study explored the strengths and weaknesses of medical students’ email etiquette and factors that contribute to professional email writing.

**Methods:**

A total of 210 emails from four faculty members at Seoul National University College of Medicine were collected. An evaluation criteria and a scoring rubric were developed based on the various email-writing guidelines. The rubric comprised 10 items, including nine items for evaluation related to the email components and one item for the assessment of global impression of politeness. Three evaluators independently assessed all emails according to the criteria.

**Results:**

Students were identified as being 61.0 % male and 52.8 % were in the undergraduate-entry program. The sum of each component score was 62.21 out of 100 and the mean value for global impression was 2.6 out of 4. The results demonstrated that students’ email etiquettes remained low-to-mediocre for most criteria, except for readability and honorifics. Three criteria, salutation (*r*=0.668), closing (*r*=0.653), and sign-off (*r*=0.646), showed a strong positive correlation with the global impression of politeness. Whether a student entered a graduate-entry program or an undergraduate-entry program significantly contributed to professional email writing after other variables were controlled.

**Conclusions:**

Although students in the graduate-entry program demonstrated a relatively superior level of email etiquette, the majority of medical students did not write emails professionally. Educating all medical students in email etiquette may well contribute to the improvement of student–faculty relationships as well as their email writing.

## Background

Email has consolidated its status as an indispensable method of communication in both businesses and academia. Medicine is not an exception to this trend. Emails have become commonplace in doctors’ routine work, including communicating with their patients, obtaining consultations from other specialists, and collaborating with other researchers on scholarly projects [1]. Similarly, emails are commonly used in medical education as the usual method of communication between faculty members and students and sometimes they are even developed as a teaching tool to cultivate communication skills or professionalism [2, 3].

Practical advantages to using email communication for both faculty members and students have been suggested in the literature. Not only does email help overcome time and space restraints when it is not possible to have a face-to-face meeting, it also creates opportunities for interactions outside as well as inside the classroom [4]. Other than these practical advantages, some researchers have also reported that there are educational benefits and learning experiences such as quality improvement of the relationships and feedback enhancement between students and faculty members [5].

However, there is mounting tension in colleges as the amount of email increases. Unprofessional emails from students have become a social issue [6], which was proven by researchers who demonstrated faculty discontent about students’ email communications lacking formality [7, 8]. Literature about the evaluation of actual emails based on published email etiquette guidelines suggests that students do need to improve their email communication [8, 9]. An email etiquette disagreement between students and faculty members worsens the situation; regardless of age, faculty members are more bothered than students are when they encounter an email with a low degree of formality [8]. Additionally, an unprofessional email does not only induce unpleasant feelings, it also damages the credibility of the email, lowers the willingness of faculty members to help or collaborate with students, and even leads the faculty members to underestimate the competency of the student who sent the email [8, 10].

Considering the distinctive nature of medical education, a faculty member’s negative perception of a student may have a more adverse effect than in other areas of higher education. For example, despite the increasing importance of direct engagement in patient care during clinical clerkship [11], a faculty member might be reluctant to include a student in a team if he or she has a negative perception of the student’s competency. Moreover, because a considerable proportion of students tend to have residency training in the hospital affiliated with the medical school that he or she graduated from [12], the relationship between students and faculty members may persist beyond undergraduate medical education to graduate medical education.

With the growing use of email, the potential negative influences from unprofessional emails, and the distinctive characteristics of student–faculty relationships in medical education, the importance of proper etiquette in email writing cannot be emphasized enough to medical students. However, to the best of our knowledge, studies on medical students’ email etiquette are scarce. Although literature about the perception of professionalism in medical students’ online posts exist [13, 14], these results cannot simply be applied to email writing because emails, which have a specific purpose and receiver, are different from online posts, which are often multifaceted with a relatively unspecified audience.

Moreover, while a scarcity of studies limits knowledge of the current status of medical students’ general email writing and etiquette, the cultural and lingual context of South Korea as an East Asian country is another subtle hurdle. Ways of expressing and interpreting politeness can differ depending on culture [15], which is reflected in email communication [16]. For example, people in South Korea (considered a high-context culture) are found to be “more socially oriented, to be more confrontation-avoiding, and to have more trouble dealing with new situations” in their communication compared with people in the low-context culture of U.S. society [17]. In addition, a cross-cultural comparison study showed that Koreans felt uncomfortable addressing others by their first name instead of their title, which also could be attributable to the high-context Korean culture preferring greater formality in email communication [16]. Given these differences, existing email-writing and etiquette guidelines need to be reviewed and revised for application in the cultural and lingual context of South Korea.

This study has the following aims: to find the strengths and weaknesses of medical students’ email etiquette by assessing and analyzing their emails; and to identify students’ characteristics that contribute to the maintenance of proper email etiquette. Based on our results, we will discuss what sort of education may be needed to improve email etiquette and how it could be achieved.

## Methods

### Setting and samples

This study was carried out in Seoul National University College of Medicine (SNUCM), which operates two Medical Doctor (MD) programs simultaneously. One is an undergraduate-entry program (UEP) including 6 years of medical education, which comprises a 2-year premedical course and a 4-year medical course. The other is a graduate-entry program (GEP). Students who enter the GEP take an identical 4-year medical course to the UEP students.

A total of 210 emails written by SNUCM medical students from 2012 to 2015 were studied. The emails were collected by requesting email messages from eight SNUCM faculty members who were internally recognized as active participants in various formal and informal undergraduate medical education programs. Two were in charge of the premedical course, two were mainly responsible for basic medical science education, and the remaining four were clinical faculty members. Two faculty members in the premedical course and two from clinical specialties agreed to provide emails stored in their inbox.

### Criteria development

To develop criteria for email evaluation, we first reviewed the previous literature including guidelines that deal with email writing and etiquette [18–20] and studies evaluating college students’ emails [8, 9] or dealing with workplace email writing [21]. Based on the review, two of the authors (DHK and JJY) developed criteria and specific items for email evaluation through an iterative process that embraces commonly found errors in the students’ email as well as general email-writing guidelines (Table 1). A panel of experts reviewed the validity evidence of the items according to the concepts suggested by Downing [22]. First, to ensure content validity, items were reviewed to check whether they unbiasedly represented general email components. Second, regarding response process-related validity evidence, the panel decided on the evaluation scale (a two- or three-point scale) of each criterion by reviewing the existing literature and possible politeness classifications for various expressions. Third, item–item correlation and item–total correlation were calculated after evaluation to identify the internal structure of criteria. Besides, the correlation of total score with the global impression of politeness (Q10) was also analyzed. During the process of criteria development, whenever there was any disagreement, the authors and the expert panel discussed the subject until they reached consensus.

**Table 1:** Data collected on students and their emails

Categories			Specific parameters	Possible entries / Point
General information (Student)			Course (at the time mailed)	PM, M
			Gender	Male/Female
			Year of birth	4-digit # (YYYY)
			Admission type	UEP, GEP
General information (Email)			Email date	8-digit # (YYYYMMDD)
			Email initiated by a student	Yes / No^a)^
			Email account provider	SNU email (@snu.ac.kr)
				Non-SNU email (other than @snu.ac.kr)
Email evaluation criteria	Subject line (Q1, Q2)	Q1. Clarity and conciseness	Clear and concise description of the purpose	+2
			Irrelevant subject line^b)^	+1
			No subject	0
		Q2. Name of the subject	States the name of a curriculum subject	+1 (Checklist, add point)
	Message body (Q3–Q8)	Q3. Salutation	Greetings and Dear Dr./Professor	+2
			Greetings or Dear Dr./Professor	+1
			No salutation	0
		Q4. Self-identification ^c), d)^	Class level and SIN	+2
			Class level or SIN	+1
			None of the two	0
		Q5. Readability	Having sufficient readability or comprehensibility^e), f)^	+1 (Checklist, add point)
		Q6. Use of honorifics	Using proper honorifics throughout the email ^d)^	+1 (Checklist, add point)
		Q7. Use of internet slang	Not using internet slang such as ungrammatical abbreviations or emoticons^d)^	+1 (Checklist, add point)
		Q8. Closing remarks	Including proper closing remarks	+1 (Checklist, add point)
	Sign-off (Q9)		Name and complimentary closing	+2
			Name only	+1
			No signature or self-identification	0
	Global impression of politeness (Q10)			1 (very impolite) – 4 (very polite)

^a)^“No” indicates that the email was sent in reply to the faculty members’ email; ^b)^ This includes the name of the sender, salutation, or greetings. ^c)^These criteria can be skipped if an email is sent in reply to an email from a faculty; ^d)^The subject line as well as the message body was subjected to evaluation; ^e)^An email with “no message body” gets 0 points; ^f)^Evaluate grammatical or spelling errors and whether paragraphs are separated properly

*PM* premedical course, *M* medical course, *UEP* undergraduate-entry program, *GEP* graduate-entry program, *SNU* Seoul National University

The criteria consisted of 10 items, which includes nine items (Q1 to Q9) allotted for the evaluation of each part of an email (two for the subject line, six for the message body, and one for sign-off) and one item (Q10) that intended to evaluate the global impression of the politeness of an email. Items Q1 to Q9 were graded with either a two- or three-point scale according to the given rubric. For the Q10 item, evaluators were asked to evaluate the global impression of politeness that he or she felt when reading the email on a 4-point Likert scale from 1 (very impolite) to 4 (very polite), regardless of the scores of the nine previous items.

We tried to collect information from the emails and their senders to achieve the second aim of our study. Student information (age, gender, and admission type) was derived from the student database by using the stated or revealed information in an email. Therefore, some information was not collectable from some students, such as those who did not specify either their name or student identification number (SIN), or they did not send emails from the university-provided email account, and we inevitably excluded these students during our further analysis using this information.

### Email evaluation

Three evaluators who were medical education professionals participated in the email evaluation. Before conducting the actual evaluation, they were provided with an explanation of the criteria standards and meanings, specific evaluation methods, and five sample cases to which the criteria was applied. To assure the evaluation reliability, experiential training was performed with 20 randomly selected emails, which corresponded to 10 % of all emails. The evaluators first performed their evaluation independently, then they discussed their score disagreements to minimize the individual differences. For the full-scale evaluation, evaluators were provided with hardcopies of the emails, criteria, and a common form to record the results. Prior to distribution, the emails were randomly ordered and anonymized by removing personally identifiable information, such as name, email address, and SIN. Evaluation was confined to the subject lines and message bodies, and the attached files, including their names, were excluded from evaluation.

### Dependent and independent variables

In this study, dependent variables were the mean values of the three independent evaluators’ scores for each criterion. However, to compare total scores between emails, a raw score (i.e., simple sum of items) had to be converted into a percentage score by dividing it by the maximum attainable score of an email because each email had different maximum attainable scores. For example, for emails that were irrelevant to the education program or courses, Q2 cannot be evaluated, which resulted in a total score of 12 or lower. For these reasons, any email with a raw score of 6 could have a different percentage score according to its maximum attainable score, such as 46.2 %, 50.0 %, and 60.0 % for the maximum attainable scores of 13, 12, and 10, respectively.

Six independent variables were explored in this study, course, gender, age at sending the email, admission type, email account provider, and whether the email is an initiator or not. For the course variable, students were divided into premedical and medical groups according to their course at the time of sending the email. Students’ types of admission (UEP and GEP) were also included as independent variables. The age at sending was calculated by using “year of birth” and “email date.” Emails were classified into two groups according to whether an email was initiated by a student or was sent in reply to the faculty’s email. Finally, emails posted from institutionally provided account were grouped separately from those posted from noninstitutionally provided email accounts.

### Statistical analysis

Intraclass correlation coefficient (ICC, two-way mixed-effects model [absolute agreement]) was used to measure the interrater reliability of three evaluators. A *t*-test and Pearson correlation was used to find the differences in email etiquette due to the characteristics of emails or students. For the Pearson correlation coefficient, we followed Evans’ classification of the strength of correlation [23], which classified values above 0.6 as having strong correlation and below 0.4 as having weak correlation. To perform a multiple regression analysis, all six independent variables were entered as potential email etiquette predictors. Because of the continuous nature of our dependent variables (total etiquette score and global impression of politeness), linear regression was chosen instead of logistic regression which presents odds ratios but requires the dependent variables to be dichotomous. Basically, all analyses were conducted using the raw scores obtained from the original scoring rubric. However, because some analyses regarding total scores could be influenced by the scoring scale differences between criteria, identical analyses were repeated if necessary after adjusting three-point scale items (0, 1, and 2) to have equal maximum points with two-point scale items (i.e., 0, 0.5, and 1). IBM SPSS Statistics for Windows software (version 20; IBM Corp., Armonk, NY, USA) was used for all statistical analyses.

Regarding the presentation of the regression analysis, albeit there were concerns in relation to the possibility of overrepresenting the students who contributed more than one email in the sample, the focus was on “emails themselves” instead of “individual students who have their politeness averaged across emails.” The first reason is because we assumed that students’ level of email etiquette may change depending on the situation (i.e., independent variable). Therefore, simply averaging the level of politeness across emails would not be reasonable considering the possible diversity of students’ email etiquette when influenced by the context. For example, two emails from “student A” could be different from each other in terms of etiquette, if one was sent during the premedical studies (e.g., in 2013) and the other during medical studies (e.g., in 2015) because the student has aged and progressed with their studies in the meantime. Similarly, two emails from “student B” might also have been written in a different manner, if one was sent from ‘studentb@snu.ac.kr (university-provided account)’ and the other from ‘studentb@hotmail.com (personal/private account).’ Second, contrary to the continuous variables such as email etiquette score and age, which can be averaged, we assumed that it may not be appropriate to produce an average of nominal variables, such as course (premedical vs medical) and email account used (university-provided vs personal).

## Results

### Student and email characteristics

A total of 210 emails were provided by four faculty members. Ninety percent of the emails initiated an email thread. Thirty percent were sent using the Seoul National University-provided email account (Table 2). Based on the information given in an email, such as name, SIN, and email address, 159 different senders were identified from the student database. Their average age was 23.1 years and 47.1 % of the students were male. Among students who sent emails during their medical course, 66 students were identified as UEP students and 59 students were identified as GEP students.

**Table 2:** Descriptive statistics for emails and their senders

			Total sample (100 %, *N* = 210)
Emails	Receiver (Professor)	A	71.4 % (150)
		B	11.0 % (23)
		C	10.0 % (21)
		D	7.6 % (16)
	Year of sending	2012	18.6 % (39)
		2013	40.0 % (84)
		2014	36.2 % (76)
		2015	5.2 % (11)
	Place in a thread	Initiator	90.0 % (189)
		Reply	10.0 % (21)
	Email account provider	SNU mail (@snu.ac.kr)	30.0 % (63)
		Non-SNU (other than @snu.ac.kr)	70.0 % (147)
Students	Age		Mean 23.06 years
			(SD 2.42; 18.3–30.8)
	Gender	Male	47.1 % (99)
		Female	30.0 % (63)
		Not identifiable	22.9 % (48)
	Course (point of sending)	Premedical	17.6 % (37)
		Medical	82.4 % (173)
	Admission type	UEP	38.2 % ^a)^ (66)
		GEP	34.1 % ^a)^ (59)
		Not identifiable	22.7 % ^a)^ (48)

^a)^For admission type, each proportion was calculated by using the number of students who sent emails during their medical course (173) as a denominator

*UEP* undergraduate-entry program, *GEP* graduate-entry program, *SNU* Seoul National University

### Descriptive analysis of the individual items

ICC, interrater reliability, of the total score was 0.905 and that of Q10 (global impression of politeness) was 0.707 (Table 3). The ICCs of the criteria was mostly near 0.8 or above, except for Q5 (Readability, ICC=0.430) and Q6 (Honorifics, ICC=0.387). This finding could be explained by the fact that few students were assessed as being incompetent in achieving readability and using proper honorifics in email writing, which could be supported by the finding that the average score of these items were as high as 0.94 out of 1.

**Table 3:** Email evaluation and intraclass correlation results

Categories	Subject		Message body						Sign-off (Q9)	Global impression of politeness (Q10)	Total Score
	(Q1, Q2)		(Q3–Q8)								
	Clarity and conciseness	Subject name	Salutation	Self-identification	Readability	Honorifics	Internet slang	Closing remarks			
Maximum points	2	1	2	2	1	1	1	1	2	4	100
Average	1.46	0.5	1.22	0.63	0.94	0.94	0.67	0.76	0.95	2.6	62.21
SD	0.48	0.48	0.91	0.5	0.18	0.18	0.45	0.4	0.97	0.8	17.55
Intraclass correlation	0.875	0.889	0.976	0.834	0.430	0.387	0.862	0.793	0.956	0.707	0.935

On average, the total email etiquette scores were 62.21 out of 100 and the scores of the global impression of politeness were 2.6 out of 4. Not only were the global impressions mediocre, the total scores demonstrate that emails did not sufficiently comply with most of the individual criteria either. For instance, subject lines, whose score was 1.46 out of 2 on average, were not completed with the purpose or key idea of an email. Emails also easily lacked the name of the subject with which the email was concerned. In terms of self-identification, few students specified themselves by stating both their class level and SIN. The average score was as low as 0.63 as a result. Salutations were often incomplete or even omitted. Students were divided into two types of sign-off, those who omit it and those who include the complete form; the average score was 0.95 (Table 3).

In terms of internal structure, all Pearson correlation coefficients between items Q1 to Q9 were significant but weak and ranged from 0.146 to 0.349. The global impression of politeness score showed a strongly significant correlation with salutation, closing remarks, and sign-off while two subject-line items did not show any significant correlation (Table 4). The total score was positively correlated with all criteria, while strengths differed depending on the item. Eight out of nine items, except Q1, maintained their positive correlation with total score when raw scores were converted by equalizing the maximum points of all criteria.

**Table 4:** Correlations between global impression of politeness, total score, and each criteria

Variable		Subject		Message body						Sign-off	Total Score
		(Q1, Q2)		(Q3–Q8)						(Q9)	
		Clarity and conciseness	Subject name	Salutation	Self-identification	Readability	Honorifics	Internet slang	Closing remarks		
Global impression of politeness	Correlation	−0.083	−0.005	0.668	0.297	0.167	0.325	0.148	0.653	0.646	0.833
	*p*-value^a)^	0.258	0.943	<0.001	<0.001	0.016	<0.001	0.033	<0.001	<0.001	<0.001
Total score	Correlation	0.197	0.185	0.650	0.441	0.159	0.275	0.325	0.481	0.703	n.a.
	*p*-value^a)^	0.007	0.013	<0.001	<0.001	0.022	<0.001	<0.001	<0.001	<0.001	

^a)^Pearson correlation

*n.a.* not available

### Univariate analyses

We examined how medical school students differ in their email etiquette depending on the sender and email characteristics (Table 5). As a student’s age increases, both global impression and total scores were improved simultaneously, but their correlation strengths were weak regardless of maximum score adjustment. For most criteria, no statistically significant difference between male and female students was identified when we conducted a univariate analysis; however, emails from medical course and GEP students tended to give a more positive impression and follow email etiquette more closely than did emails from premedical course and UEP students.

**Table 5:** The influence of various factors on email etiquette (univariate analysis)

Variable		Subject		Message body						Sign-off (Q9)	Global impression of politeness (Q10)	Total score
		(Q1, Q2)		(Q3–Q8)								
		Clarity and conciseness	Subject name	Salutation	Self-identification	Readability	Honorifics	Internet slang	Closing remarks			
Age at sending (*n* = 162)	Correlation^a)^	0.061	0.043	0.034	−0.063	0.202*	−0.080	−0.050	0.158	0.321	0.228	0.206
	*p*-value	0.469	0.626	0.664	0.449	0.010	0.316	0.531	0.045	<0.001	0.003	0.009
Gender (*n* = 140)	Male (87)^b)^	1.51 ± 0.48	0.53 ± 0.48	1.1 ± 0.95	0.71 ± 0.5	0.95 ± 0.17	0.96 ± 0.13	0.8 ± 0.38	0.71 ± 0.42	0.85 ± 0.98	2.57 ± 0.84	62.59 ± 17.2
	Female (53)^b)^	1.54 ± 0.49	0.52 ± 0.48	1.38 ± 0.85	0.59 ± 0.52	0.94 ± 0.19	0.91 ± 0.23	0.54 ± 0.48	0.78 ± 0.37	0.97 ± 0.96	2.6 ± 0.78	63.21 ± 18.51
	*p*-value^c)^	0.742	0.936	0.057	0.158	0.891	0.099	0.001	0.330	0.438	0.775	0.829
Current course (*n* = 210)	Premedical (37)^b)^	1.61 ± 0.45	0.45 ± 0.49	1.19 ± 0.97	0.82 ± 0.49	0.87 ± 0.26	0.97 ± 0.12	0.7 ± 0.43	0.63 ± 0.43	0.41 ± 0.79	2.31 ± 0.77	57.16 ± 20.35
	Medical (173)^b)^	1.43 ± 0.48	0.51 ± 0.48	1.23 ± 0.9	0.59 ± 0.5	0.96 ± 0.16	0.93 ± 0.19	0.67 ± 0.45	0.78 ± 0.39	1.06 ± 0.97	2.66 ± 0.8	63.3 ± 16.76
	*p*-value^c)^	0.062	0.563	0.818	0.023	0.051	0.118	0.670	0.036	<0.001	0.013	0.053
Admission type (*n* = 125)	UEP (66)^b)^	1.47 ± 0.49	0.52 ± 0.49	1.04 ± 0.94	0.59 ± 0.5	0.95 ± 0.15	0.9 ± 0.23	0.77 ± 0.4	0.69 ± 0.44	0.78 ± 0.94	2.37 ± 0.78	59.61 ± 15.3
	GEP (59)^b)^	1.53 ± 0.48	0.59 ± 0.46	1.42 ± 0.84	0.67 ± 0.51	0.98 ± 0.12	0.97 ± 0.11	0.61 ± 0.47	0.86 ± 0.3	1.34 ± 0.93	2.99 ± 0.71	69.99 ± 16.2
	*p*-value^c)^	0.563	0.452	0.016	0.404	0.366	0.026	0.048	0.009	0.001	<0.001	<0.001

^a)^Pearson correlation; ^b)^Mean ± SD ^c)^independent sample t-test

*UEP* undergraduate-entry program, *GEP* graduate-entry program

### Multivariate analyses

When the variables were controlled by a multiple regression analysis, the type of entry program was the only factor that significantly influenced global impressions of politeness (*p* < 0.001) and total scores (*p*=0.003) (Table 6). When the total scores were recalculated after equalizing all maximum points, in addition to “Admission to a GEP” (95 % CI=1.685, 13.204; *p*=0.012), initiator emails had lower total scores by 6.851 points (95 % CI=−12.829, −0.873; *p*=0.025) than reply emails.

**Table 6:** Influence of various characteristics of emails and senders on email etiquette (multiple regression)

Variable		Unstandardized coefficient		Standardized coefficient	*t*	*p*-value
		B	Standard error	β		
Global impression of politeness	(Constant)	3.042	0.907		3.352	0.001
	Current class (Medical)	0.160	0.203	0.083	0.789	0.431
	Initiator	0.090	0.182	0.037	0.493	0.623
	Email provider (SNU email)	0.033	0.140	0.019	0.237	0.813
	Gender (Female)	−0.072	0.126	−0.043	−0.571	0.569
	Graduate-entry program	0.723	0.176	0.430	4.112	<0.001
	Age at sending the email	−0.036	0.044	−0.108	−0.824	0.411
Total score	(Constant)	66.961	20.353		3.290	0.001
	Current class (Medical)	3.494	4.549	0.083	0.768	0.444
	Initiator	2.313	4.091	0.044	0.565	0.573
	Email provider (SNU email)	0.117	3.130	0.003	0.038	0.970
	Gender (Female)	−1.229	2.821	−0.034	−0.436	0.664
	Graduate-entry program	11.983	3.941	0.327	3.040	0.003
	Age at sending the email	−0.500	0.988	−0.069	−0.506	0.613

## Discussion

In this study, we examined the email etiquette and factors affecting medical students’ communication with faculty members. The results indicate that many medical students lack email etiquette for many email components, which is consistent with previous studies conducted in other colleges and disciplines. Whether a student was admitted in the GEP or UEP was the significant predictor of the proper following of email etiquettes as well as leaving positive impressions on faculty members.

One of the major findings of the study is that medical students were not sufficiently qualified as professional email writers. The total email etiquette scores were 62.21 out of 100 and the global impression of politeness scores were 2.6 out of 4. Researchers have already shown that being a so-called “digital native” is an insufficient condition for professional email writing [3]. The results of this study are comparable with previous literature, such as one that evaluated introductory biology class students and demonstrated their email format scores to be less than eight points out of 13 [9]. Another study of medical students’ email in a simulated patient communication situation showed scores of about 14.36 out of 18, which is relatively higher but still unsatisfactory [2]. Put together, these studies show that despite the variety of settings or evaluation criteria, college students, including medical students, need to improve their writing skills to write email professionally.

Among the criteria, readability and honorifics were the only two that showed sufficient levels, with average scores as high as 0.94 for both. An email with a distracting background or using a difficult-to-read font is unpleasant for receivers to read most of the time [24]; therefore, it is positive that there were very few emails that were sent in this kind of style. In addition, although it is common that a word may change its form completely in Korean honorifics, hardly any students made honorific errors. This finding might be attributed to the high admission standards that require medical school applicants to achieve high scores in “Korean language and literature.”

However, evaluation results were not satisfactory for other criteria. Subject lines often included just salutations or greetings to the professor, with an average score of 1.46 out of 2. A properly written subject line that meets its own purpose is a concise and clear summary of the aim and contents of the email, which helps the receiver to classify or judge the priority of an email before opening it [25]. Nonetheless, the majority of medical students showed their lack of understanding of the function of a subject line by using it as a space for salutation or stating their name and giving no clues to the forthcoming contents to receivers, which is considered inappropriate [26]. Following this lack of understanding among students, proper use of the subject line should be emphasized because it is consistently argued for and included in most of the guidelines [1, 18], unlike other items, such as recommendations on the use of Internet slang or emoticons, which are relatively inconsistently expressed depending on the guideline [18].

Students’ tendency to omit information about their topic from the subject line is also worth examining. Students are recommended to include the topic of their email in the subject line when it is relevant to their educational setting [9]. Clear subject lines are important not only for faculty members, but also for students because they may prevent any possible confusion or miscommunication when they are dealing with subject-specific issues like tests, assignments, or grades. Many faculty members are usually involved in educating first- to fourth-year students, so even a seemingly trivial query about a disease or specialty may not receive the expected response if a faculty member replies without having information about the student who asked the question.

In particular, the way students identified themselves needed improvement as shown by the two lowest scored items, self-identification and sign-off, which are directly related to introducing oneself. Students are prone to leave out their basic identifying information such as class level or SIN, which suggests that students misperceive the educational environment. When students send an email, for instance, they might unconsciously think that stating their name is sufficient for the faculty member to identify the student. This could be a manifestation of the oft-mentioned egocentric and insensitive nature of medical students [27], because from the faculty members’ perspective, identifying a student by only his or her name is not only inefficient, but also sometimes impossible because different students may have the same name.

Criteria varied in their relationship with global impression of politeness. The previous literature has highlighted the importance of salutation and sign-off [24]. Salutation is recognized as the key indicator that demonstrates the politeness, status, and social distance of an email [19]. It is consistent with the study results, which have shown a strongly positive correlation between the global impression of politeness and three criteria: salutation (*r*=0.668), closing (*r*=0.653), and sign-off (*r*=0.646). Their strongly positive correlations may be attributed to the receiver’s first and last impressions being influenced by their locations in an email, but further investigation on how and what other factors contribute may be needed.

In multivariate analysis, only admission to the GEP remained the single consistent predictor for professional email writing regardless of dependent variables. Although the regression analyses were basically focused on “emails themselves” instead of “individual students who have their politeness averaged across emails” as stated, even when the analysis was conducted in the perspective of the latter by averaging values across emails, the results were consistent; i.e., “admission to the GEP” was the single significant factor. This is not surprising given the majority of students included in this study contributed only one email each and the number of emails collected per student remained as low as 1.3 in total.

Studies comparing the academic performance between GEP and UEP students in medical education have shown that GEP students outperform on a small scale, but the probability of the contribution of older age has simultaneously also been identified [28, 29]. In our study, admission in the GEP still made a significant contribution after controlling for age, at least in respect to email communication. This might be explained by some relative strength of GEP students. Assuming that GEP students are more mature and motivated than UEP students [30], they might have paid more attention to their relationship with faculty members from the long-term perspective of their career as a doctor. Moreover, the higher self-awareness and self-control [31, 32] of GEP students may have led to this difference because they more often write their emails from a receiver-centered perspective.

### Implications for education

The findings of this study support the necessity of education about email etiquette for medical students. Researchers have already argued for email etiquette education targeting future health-care professionals because email becomes unavoidable in diverse areas of health care and medicine [25]. More importantly, it is more realistic for medical educators to assume that medical students’ email etiquette will not spontaneously develop with age. This assumption was supported by a previous study that found that students did not naturally acquire communication skills just by completing a routine educational experience [2]. Furthermore, the impact of email etiquette education is also expected to be favorable because even short training on email writing turned out to have a considerable impact on students [2, 9].

In the implementation of an education program for enhancing email etiquette, the fundamentals of email writing and etiquette need to be taught first. These fundamentals describe email components and the minimal standards required for each component. If correlations between criteria were generally weak, it would be better to teach email etiquette in components. If students find that following the guidelines closely seems excessive, they can be advised to be aware that at the very least they can avoid giving a negative impression to faculty members by ensuring that they use the proper salutation, add closing remarks, and include a suitable sign-off. Regarding educational methods, it is worth mentioning that faculty members are usually passive in giving explicit feedback on their impression of students from the email communication [33]. Feedback is most effective when provided near the time of the communication and when based on direct observation [34]; therefore, informal teaching, such as faculty including feedback in their email response to students, would be most effective if performed immediately.

Alternatively, the primary reason for unprofessional email writing may not essentially be a lack of knowledge or skill in writing. Instead, it could be matter of “attitude.” If this is the case, it might be effective to inform students about how their unprofessional emails could develop into potential risks for the students, such as faculty members’ negative feelings, lowered possibility of a response, and even underestimation of the personality and intelligence of the senders [24]. Similarly, existing literature about online posting has pointed out that the “concept of personal risk” is central when judging whether an online posting is inappropriate [13].

Besides the necessity of email etiquette education, our findings suggest the potential for using a global rating scale instead of a checklist for evaluating email etiquette. Checklists and global ratings have been acknowledged to have different merits depending on the evaluators or the subject of evaluation [35]. However, considering the high level of correlation between the total score and the global impression of politeness, global ratings may be more valid as well as more efficient than using a lengthy checklist.

### Limitations

There are some limitations to this study. First, this study was conducted in a single South Korean institution and the quality and composition of the samples could have been affected by curricular, organizational, or sociocultural characteristics. Nevertheless, the major study settings, such as “medical school,” “digital-native students,” or “email communication with professor,” are common settings in many countries. Second, the linguistic traits of Korean may have been reflected in some details of the evaluation criteria, which may possibly limit the generalizability of the study to other languages or cultures. It remains to be seen whether similar results are observed in other countries or cultures, although we observed many shared similarities in Korean and English email etiquette during the criteria development. Third, reference groups are needed for more accurate interpretation of this study. For example, it remains to be seen whether college students who major in other disciplines show similar professional email-writing ability. Further studies in medical schools in other countries are also necessary to understand the weaknesses of South Korean medical students more precisely and even beyond cultural context. Fourth, there is no absolute “right answer” for appropriate email etiquette even when criteria are comprehensive and generalizable because email is a so-called “written conversation” with the features of both spoken and written languages [36]. The recommendation disagreements between guidelines on a similar issue can be understood in this context [18]. Nonetheless, we tried to apply the guidelines rather conservatively for evaluation because it may be considered “safer” to follow a conservative guideline given that a high degree of formality is recommended, particularly for work-related emails [1].

## Conclusions

In this study, we analyzed medical students’ emails to faculty members to examine their strengths, weaknesses, and factors related to writing emails with proper etiquette. When compared with general guidelines, medical students’ emails were not formal enough except for a few criteria. “Admission to a GEP” was the sole predictor for professional email writing. While this study is limited by the linguistic and cultural traits of South Korea, it seems evident that email writing and etiquette education is required for medical students. Email etiquette in other countries or that of students using other languages needs to be examined in future research.

### Ethics approval

This study was approved by the Institutional Review Board of SNUCM. After considering the nature of the study, the Institutional Review Board waived the need for informed consent in the ethical review process.

### Availability of data and materials

Data will not be shared due to restrictions stipulated by the Institutional Review Board of SNUCM that approved this study.

## Abbreviations

GEP: Graduate-entry program
ICC: Intraclass correlation coefficient
MD: Medical Doctor
SIN: Student identification number
SNU: Seoul National University
SNUCM: Seoul National University College of Medicine
UEP: Undergraduate-entry program
